# Observing third-party ostracism enhances facial mimicry in 30-month-olds

**DOI:** 10.1016/j.jecp.2020.104862

**Published:** 2020-08

**Authors:** Carina de Klerk, Hannah Albiston, Chiara Bulgarelli, Victoria Southgate, Antonia Hamilton

**Affiliations:** a Centre for Brain and Cognitive Development, Birkbeck College, University of London, London WC1E 7HX, UK; b Department of Psychology, University of Essex, Colchester, Essex CO4 3SQ, UK; c Department of Psychology, University of Bath, Claverton Down, Bath BA2 7AY, UK; d Department of Medical Physics and Bioengineering, University College London, London WC1E 6BT, UK; e Department of Psychology, University of Copenhagen, 1353 København, Denmark; f Institute of Cognitive Neuroscience, University College London, London WC1E 6BT, UK

**Keywords:** Mimicry, Affiliation, Priming, Ostracism, Toddlerhood, Imitation

## Abstract

Mimicry is suggested to be one of the strategies via which we enhance social affiliation. Although recent studies have shown that, like adults, young children selectively mimic the facial actions of in-group over out-group members, it is unknown whether this early mimicry behavior is driven by affiliative motivations. Here we investigated the functional role of facial mimicry in early childhood by testing whether observing third-party ostracism, which has previously been shown to enhance children’s affiliative behaviors, enhances facial mimicry in 30-month-olds. Toddlers were presented with videos in which one shape was ostracized by other shapes or with control videos that did not show any ostracism. Before and after this, the toddlers observed videos of models performing facial actions (e.g., eyebrow raising, mouth opening) while we measured activation over their corresponding facial muscles using electromyography (EMG) to obtain an index of facial mimicry. We also coded the videos of the sessions for overt imitation. We found that toddlers in the ostracism condition showed greater facial mimicry at posttest than toddlers in the control condition, as indicated by both EMG and behavioral coding measures. Although the exact mechanism underlying this result needs to be investigated in future studies, this finding is consistent with social affiliation accounts of mimicry and suggests that mimicry may play a key role in maintaining affiliative bonds when toddlers perceive the risk of social exclusion.

## Introduction

It is a common phenomenon during social interactions: without noticing, we copy our interaction partner's behavior or accent. This tendency to spontaneously and unconsciously copy or “mimic” others’ behaviors has been shown to play an important role in enhancing social affiliation. For example, it contributes to the development of liking and rapport between strangers and makes social interactions more smooth and enjoyable (for reviews, see Chartrand and Lakin, 2013, Duffy and Chartrand, 2015, van Baaren et al., 2009). Although this mimicry behavior is typically not under conscious control, it has been suggested that it is nevertheless strategically employed to enhance social affiliation (Wang & Hamilton, 2012). This theory suggests that we anticipate the positive consequences of mimicry at some level and, therefore, will increase our mimicry whenever it is in our benefit to make others like us. Consistent with this idea, studies have shown that giving participants the goal to affiliate increases mimicry behaviors (Lakin & Chartrand, 2003). Additionally, adult participants have been shown to increase mimicry toward people they like and in-group members, whereas mimicry of out-group members is inhibited (for reviews, see Chartrand and Lakin, 2013, van Baaren et al., 2009). Mimicry has also been found to be enhanced in participants who were excluded during a Cyberball game, suggesting that mimicry may serve as a means to restore affiliation after experiencing ostracism - i.e. exclusion or rejection from the group (Cheung et al., 2015, Lakin et al., 2008).

Despite the important role mimicry is thought to serve in maintaining affiliative bonds, until recently surprisingly little was known about the development of this phenomenon. While reports of neonatal imitation of facial actions (e.g., Meltzoff & Moore, 1977) have been subject to criticism and doubt (e.g., Jones, 2009, Meltzoff et al., 2018, Oostenbroek et al., 2016), recent studies that used a more objective measure of mimicry, electromyography (EMG), have shown that facial mimicry is present from at least 4 months of age (de Klerk et al., 2018, Isomura and Nakano, 2016, Kaiser et al., 2017). Moreover, recent studies have shown that, like adults (Bourgeois & Hess, 2008), infants and school-aged children selectively mimic the facial actions of in-group members over out-group members (de Klerk et al., 2019, van Schaik and Hunnius, 2016). van Schaik and Hunnius (2016) used a minimal group paradigm (Tajfel, Billig, Bundy, & Flament, 1971) in which 3- to 6-year-old children were allocated to novel groups based on their preferred T-shirt color. They found that 4- to 6-year-olds, but not 3-year-olds, showed greater behavioral mimicry of the minimal in-group member compared to the minimal out-group member. In the adult mimicry literature, selective mimicry of in-group members is usually thought to reflect the desire to affiliate (e.g., Hess and Fischer, 2014, van Baaren et al., 2009). Therefore, one interpretation of these findings by van Schaik and Hunnius (2016) is that the children had a greater motivation to affiliate with the minimal in-group member compared to the out-group member, and mimicking her actions functioned as a means to communicate their similarity to the model (van Baaren et al., 2009). Alternatively, the in-group members may have captured the children’s attention to a greater extent, leading to increased encoding of the models’ actions and resulting in greater mimicry. The fact that there were no differences in visual attention to the in-group videos compared to the out-group videos seems to speak against this latter interpretation (van Schaik & Hunnius, 2016). However, without directly manipulating affiliative motivations, this study cannot provide conclusive evidence that such motivations underlie young children’s mimicry behaviors.

In the current study, we aimed to investigate whether young children use facial mimicry as a strategy to enhance social affiliation by manipulating their affiliative motivations and assessing how this affects their tendency to mimic others’ facial actions. We focused on 30-month-olds because this is the earliest age at which we found selective mimicry of in-group members in a minimal group paradigm (de Klerk, Bulgarelli, Hamilton, & Southgate, in preparation). To manipulate the toddlers’ affiliative motivations, we presented them with videos in which one shape was ostracized by a group of other shapes or with control videos that did not show any ostracism (stimuli from Over & Carpenter, 2009). Previous studies that used the same priming stimuli as we used here suggest that the third-party ostracism primes evoke an enhanced motivation to affiliate in 4- and 5-year-old children, as indicated by enhanced high-fidelity imitation of object-directed actions (Over & Carpenter, 2009) and drawing pictures of their friend and themselves standing significantly closer together (Song, Over, & Carpenter, 2015). The current study extends these findings by investigating the effect observing third-party ostracism has on facial mimicry, thereby providing insight into the functional role facial mimicry plays in early childhood. Before and after watching the priming stimuli, toddlers in the current study observed videos of models performing facial actions (e.g., eyebrow raising, mouth opening, frowning) while we measured activation over their corresponding facial muscles using EMG to obtain an index of facial mimicry. Given mimicry’s hypothesized role in enhancing social affiliation, we predicted that toddlers who had been primed with ostracism would mimic the facial actions of the models more than toddlers who had observed the control stimuli.

Although measuring facial mimicry using EMG provides several benefits (i.e., it allows us to capture subtle changes that occur during automatic facial mimicry and provides an objective and sensitive measure of mimicry), one may wonder how this mimicry relates to the overt behavioral mimicry observed in adult studies (e.g., Chartrand & Bargh, 1999). In the adult literature, facial mimicry as measured by EMG is generally placed in the same context as the spontaneous mimicry of other nonverbal behaviors such as postures and gestures (e.g., Chartrand and Lakin, 2013, Stel and Vonk, 2010, van Baaren et al., 2009). Indeed, facial mimicry and mimicry of postures, gestures, and mannerisms share many properties; they both seem to occur without conscious awareness (Chartrand and Bargh, 1999, Dimberg et al., 2000), are influenced by the same factors such as group membership (Bourgeois and Hess, 2008, Yabar et al., 2006), and are supported by similar neural mechanisms (Likowski et al., 2012, Wang and Hamilton, 2012). Furthermore, it has been suggested that the subtle mimicry that can be measured by EMG may be a building block for more overt and extended matching (Moody and McIntosh, 2006, Moody and McIntosh, 2011). It has been shown that our emotions and social perception can be influenced by facial stimuli that we cannot consciously perceive (Bornstein et al., 1987, Dimberg et al., 2000, Li et al., 2008, Sweeny et al., 2009). Therefore, even the subtle mimicry that is detected by EMG could potentially have an effect on social affiliation*.* However, one could also argue that mimicry needs to be perceptible to exert an influence on one’s social partner (Sato & Yoshikawa, 2007). Therefore, we also coded the videos of the EMG sessions to calculate the percentage of trials in which the toddlers demonstrated overt mimicry of the facial actions and predicted that this overt mimicry may be more strongly influenced by toddlers’ affiliative motivations than the mixture of overt and covert mimicry that was measured using EMG.

## Method

### Participants

A total of 56 30-month-old toddlers were brought to the lab and participated in this experiment. Of these, 35 toddlers provided sufficient data to be included in the EMG analyses (Ostracism condition: *n* = 17, 10 girls, *M*_age_ = 919 days, *SD* = 9.16, range = 906–943; Control condition: *n* = 18, 8 girls; *M*_age_ = 917 days, *SD* = 10.53, range = 897–933). A total of 21 toddlers were excluded from the EMG analyses due to the toddler refusing to wear the EMG electrodes (*n* = 1); experimenter error (*n* = 2); an insufficient number of good trials at pretest (*n* = 2) or posttest (*n* = 5); or the posttest not having been done either because the toddler did not want to watch the stimuli at pretest (*n* = 2), because the toddler was very upset by the EMG electrodes at pretest (*n* = 4), or because the toddler refused to wear the EMG electrodes a second time (*n* = 2) or to watch the stimuli a second time (*n* = 3). The percentage of excluded participants (37.5%) in the current study is lower than that in previous facial EMG research with toddlers (e.g., Geangu, Quadrelli, Conte, Croci, & Turati, 2016).

All 35 toddlers who were included in the EMG analyses plus an additional 6 toddlers^1^ were included in the overt mimicry analyses (total *N* = 41; Ostracism condition: *n* = 19, 11 girls, *M*_age_ = 918 days, *SD* = 9.71, range = 901–943 days; Control condition: *n* = 22, 10 girls, *M*_age_ = 917 days, *SD* = 10.57, range = 897–937). A total of 15 toddlers were excluded from these analyses because of experimenter error (*n* = 2), because the posttest had not been done (*n* = 11; see above for reasons), or because the toddler had an insufficient number of good trials at posttest (*n* = 2).

This study was part of a longitudinal project investigating the development of mimicry. Participants were originally recruited through the lab’s database, which includes details of families who have voluntarily signed up for participation in studies on infant development, when the children were 4 months old. Participants had been to the lab for testing sessions at 4, 11, 18, and 24 months of age before the current session, although 4 of them had started their participation at 18 months. We tested all the children who were able to come back for this fifth visit of the project. We recruited an additional 6 participants from the database to even out the number of participants who had been tested in the two conditions, but after video-coding we nevertheless ended up with a slightly uneven number of included participants in the two conditions. Power analyses using effect sizes based on the previous work by Over and Carpenter (2009) demonstrated that a total sample size of 33 participants would have provided enough power (.90 with an alpha level of .05) to identify similar effects.

All included toddlers were born full-term, healthy, and with normal birth weight. The study received approval from the institutional research ethics committee. Written informed consent was obtained from the toddlers’ caregivers.

### Stimuli and procedure

#### Pretest and posttest EMG session

Before and after the toddlers were presented with either ostracism or control videos, we measured their facial mimicry. During this pretest and posttest, the toddlers were presented with videos of two female models performing eyebrow and mouth actions (i.e., eyebrow raising, frowning, tongue protrusion, and mouth opening) while we measured activation over the frontalis region, the corrugator region, and the masseter region using EMG. The same stimuli were used when the infants were 4, 11, 18, and 24 months of age to investigate the longitudinal development of facial mimicry (these data are the topic of separate reports).^2^

**Fig. 1:**
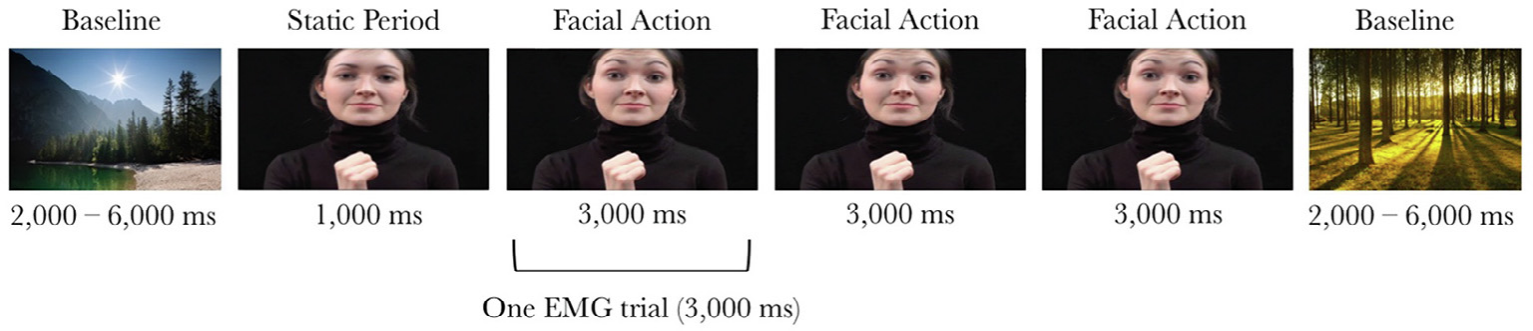
Schematic overview of the presentation of the mimicry stimuli.

The stimuli were presented on a 117-cm plasma screen, and the toddler was seated on the parent’s lap at a distance of approximately 80 cm. The stimuli were presented on a 31 × 54-cm part of the screen, and when viewed from a 80-cm distance they subtended a visual angle of 21.9° × 37.3°. Each video started with 1000 ms during which the model did not perform any actions, followed by her performing three repeats of the same facial action, each lasting 3000 ms (see Fig. 1). The 10-s videos were presented in a random order, alternated with baseline trials consisting of static pictures of houses, animals, and landscapes with a random duration of 2000–6000 ms to allow for any mimicry responses to subside before the next video was presented (see Fig. 1). If necessary, alerting sounds were played to draw the toddler’s attention back to the screen. Videos were presented until the toddler had seen approximately 12 10-s mimicry videos or until the toddler’s attention could no longer be attracted to the screen (mean number of presented videos = 13.22, *SD* = 2.4). Toddlers were video-recorded throughout the session.

The pretest EMG session was the first task the toddlers participated in, and there was approximately 60 to 90 min separating the pretest from the priming and posttest phase, during which the toddlers participated in several other behavioural tasks (e.g., a false belief task, a helping task) and neuroimaging tasks (e.g., a resting-state functional near-infrared spectroscopy recording) that were part of the bigger longitudinal study (data from these other tasks are the topic of separate reports and are not reported here). This delay was meant to ensure that the toddlers were dishabituated to the mimicry stimuli, increasing the chances that they would watch the stimuli a second time for the posttest.

The electrodes for the posttest EMG recording were applied on the toddler’s face before the Ostracism or Control condition stimuli were presented, ensuring that the posttest phase could start straight after the priming task had finished.

#### Priming task

**Fig. 2:**
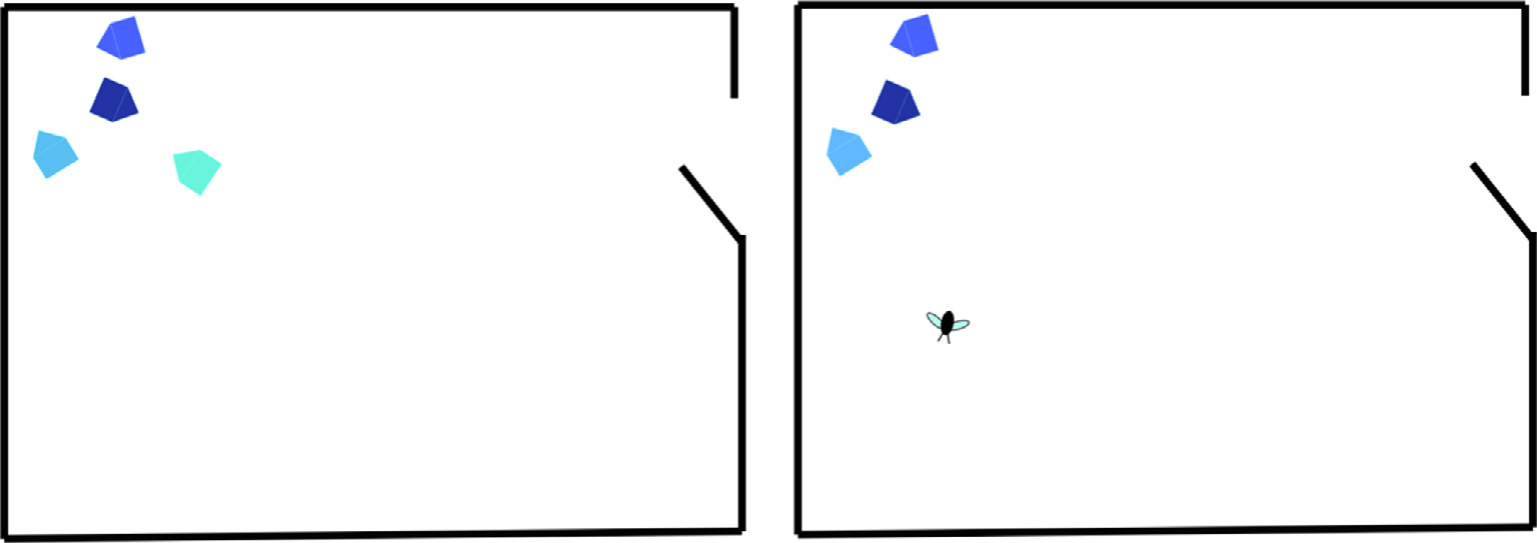
Stills from one of the Ostracism condition videos (left) and one of the Control condition videos (right).

Toddlers were randomly allocated to the Ostracism or Control condition. Toddlers in the Ostracism condition observed two short videos in which one shape was ostracized by a group of other shapes, whereas toddlers in the Control condition observed two short control videos that did not show any ostracism. The stimuli were created by Over and Carpenter (2009), using the custom animation function in PowerPoint, and were presented on a 9.7-inch tablet device (resolution of 1024 × 768) that was held at a distance of approximately 30 cm from the toddler by the experimenter (see Fig. 2 for example stills from the videos). The first video in the Ostracism condition depicted three pentagons in various shades of blue that appeared to be playing together as a group, with a fourth pentagon trying to join in but being excluded by the others. The second video in the Ostracism condition depicted two teardrop-shaped yellow objects playing together with a ball, with a third teardrop shape trying to join the game but being excluded from the subsequent ball tosses by the other two shapes. Both videos ended with the rejected shape moving away from the others and coming to a halt at the far side of the screen. The videos in the Control condition were very similar to those in the Ostracism condition but did not involve any ostracism. In these videos, the rejected shape was replaced by a different type of object (a fly in the pentagon video and a butterfly in the ball game video) that, instead of attempting to be included in the group, made random movements around the screen. These objects were approximately the same size and color as the rejected shapes in the Ostracism condition, and the number of movements they made was matched to the movements made by the rejected shapes (for more information about the stimuli, see Over & Carpenter, 2009).

#### EMG recording and processing

**Fig. 3:**
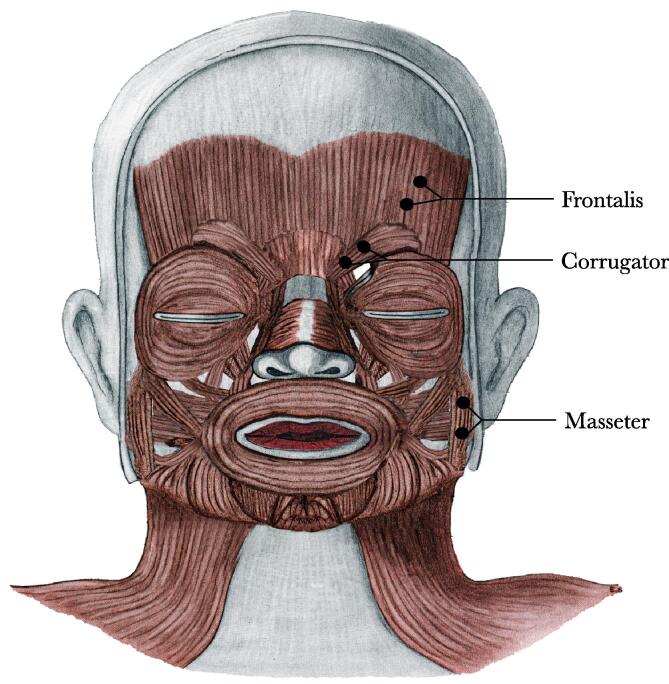
Muscle locations from which EMG activity was measured. (Image adapted by Crystal Butler with permission from E. S. Crelin’s *Functional anatomy of the newborn: An atlas* (1973), Yale University Press).

Bipolar EMG recordings were made using pediatric surface Ag/AgCl electrodes that were placed over the masseter muscle region, the frontalis muscle region, and the corrugator muscle region on the left side of the face with an inter-electrode spacing of approximately 1 cm, following recommendations by Fridlund and Cacioppo (1986) (see Fig. 3). We expected the observation of eyebrow-raising actions to be mainly associated with EMG activity over the frontalis region, frowning actions to be mainly associated with activity over the corrugator region, and mouth opening and tongue protrusion actions with activity over the masseter region. However, surface EMG electrodes measure broad nonselective firing of aggregates of motor units of muscle groups underlying and near the electrode sites (Lawrence & De Luca, 1983). Therefore, although the electrodes on the cheek would mainly have measured activity of the masseter muscle (involved in closing the mouth), they may have also picked up activity of the underlying lateral pterygoid muscles (involved in opening the mouth). Additionally, it is possible that the electrodes over the frontalis region not only recorded activity of the frontalis muscle (involved in raising the eyebrows) but also may have picked up activity of the nearby corrugator supercilii (involved in frowning) and vice versa (Fridlund & Cacioppo, 1986). Following Fridlund and Cacioppo (1986) recommendation, therefore, we use the terms *frontalis region, corrugator region,* and *masseter region* to describe EMG activity measured over these areas. The electrodes were connected to Myon wireless transmitter boxes^3^ that amplified the electrical muscle activation, which was in turn recorded using proEMG at a sampling rate of 2000 Hz. After recording, the EMG signal was filtered (high pass: 30 Hz; low pass: 500 Hz), smoothed (root mean square over 20-ms bins), and rectified (converted to absolute values).

Each 3000-ms period during which a facial action was performed by the model was treated as a separate trial (see Fig. 1). Videos were coded offline by a coder who was unaware of the experimental condition to which the toddlers had been assigned. Trials in which the toddlers did not see at least two thirds of the action, and trials in which the toddlers vocalized, smiled, cried, or had something in their mouth (e.g., their hand or their clothing), were excluded from the analyses. EMG trials in which the toddlers pulled or moved the EMG wires were also excluded. Note that, similar to previous facial mimicry research with developmental populations (e.g., Cattaneo et al., 2018, Geangu et al., 2016, Kaiser et al., 2017, Natale et al., 2014) and adult populations (e.g., Bourgeois and Hess, 2008, Likowski et al., 2008, Moody et al., 2007), we did not exclude trials based on the toddlers’ facial expressions (but see the online supplementary material for EMG analyses from which all trials with overt facial actions were removed). Only toddlers with at least 4 eyebrow action trials and 4 mouth action trials at both pretest and posttest were included in the analyses. On average, the included toddlers contributed 6.57 eyebrow-raising trials (*SD* = 3.3), 5.69 frowning trials (*SD* = 4.4), 7.00 tongue protrusion trials (*SD* = 3.7), and 6.94 mouth-opening trials (*SD* = 3.4) at pretest and 6.17 eyebrow-raising trials (*SD* = 3.4), 4.37 frowning trials (*SD* = 3.6), 6.17 tongue protrusion trials (*SD* = 3.6), and 6.63 mouth action trials (*SD* = 3.7) at posttest to the analyses. The number of included trials did not differ between the Ostracism and Control groups, *F*(1, 33) = 0.109, *p* = .743.

The EMG signal was segmented into 3000-ms epochs, and the average activity in each epoch was normalized (i.e., expressed as *z* scores by subtracting the mean and dividing by the standard deviation) within each participant and each muscle group (masseter, frontalis, and corrugator) before the epochs for each trial type were averaged together. This allows for meaningful comparison of values between muscle regions and also reduces the impact of individual differences in reactivity on the group mean. Because facial mimicry can be defined as the presence of greater activation over corresponding muscles than over noncorresponding muscles during the observation of facial actions (e.g., McIntosh et al., 2006, Oberman et al., 2009), we calculated a facial mimicry score per trial by subtracting EMG activity over the noncorresponding muscle region from EMG activity over the corresponding muscle region (e.g., when the toddler observed an eyebrow-raising trial, we subtracted activity over the masseter region from activity over the frontalis region, so that a more positive score indicates more facial mimicry). We calculated the mimicry scores as follows: (a) mimicry of eyebrow raising = EMG activity over the frontalis region − EMG activity over the masseter region, (b) mimicry of frowning = EMG activity over the corrugator region − EMG activity over the masseter region, and (c) mimicry of mouth actions = EMG activity over the masseter region − EMG activity over the frontalis region (see Fig. 3). Analyses were performed on the mean facial mimicry scores.^4^ Because of a technical issue with one of the EMG transmitter boxes, we lost the signal over the corrugator muscle on some or all the trials for a subset of the participants; this affected the data of 7 participants during the pretest (4 from the Ostracism condition) and 9 participants during the posttest (4 from the Ostracism condition). In these cases, we were unable to calculate the frowning mimicry score; thus, these trials were excluded from the analyses for these children. Because of the occasional lost signal over the corrugator muscle region, we used the activation over the frontalis muscle region (rather than the corrugator muscle region or the average of both muscles) to calculate the mouth mimicry scores.

#### Overt mimicry

Because mimicry may need to be at least minimally perceptible to exert an influence on one’s social partner, we also coded the videos of the EMG sessions for overt (i.e., visible) mimicry of the facial actions. Videos were coded offline; all trials in which the toddlers performed the same facial action as the action they were observing on the screen were given a code of 1, and trials in which they performed no facial action or a different action were given a code of 0. Eyebrow raising was defined as a vertical movement of the eyebrows, frowning was defined as a furrowing of the brow, mouth opening was defined as a lowering of the jaw, and tongue protrusion was defined as the protrusion of the tongue so that it stuck out of the mouth. Mimicry was defined as the selective activation of corresponding facial muscles; thus, instances in which toddlers performed two actions simultaneously (e.g., frowning and mouth opening) were not counted as mimicry. Because the EMG electrodes were small and placed on only one side of the toddlers’ face, overt facial actions were clearly visible on the video. The coder was unaware of the experimental condition to which the toddlers had been assigned. A subset of 20% of the videos was coded by a second coder blind to the purpose of the experiment and experimental conditions, and inter-coder reliability was very high (Cohen’s kappa = .84). Trials in which the toddlers did not see at least two thirds of the action and trials in which the toddlers were crying were excluded from the analyses. Only toddlers with at least 4 eyebrow action trials and 4 mouth action trials at both pretest and posttest were included in the analyses. On average, the included toddlers contributed 15 eyebrow action trials (*SD* = 3.4) and 15.6 mouth action trials (*SD* = 3.5) at pretest and 14.5 eyebrow action trials (*SD* = 4.4) and 15.6 mouth action trials (*SD* = 4.8) at posttest to the overt mimicry analyses. The number of included trials did not differ between the Ostracism and Control groups, *F*(1, 39) = 0.405, *p* = .528. We created an overt mimicry score by calculating the percentage of observed trials in which the toddlers demonstrated overt mimicry.

## Results

### Electromyography

**Fig. 4:**
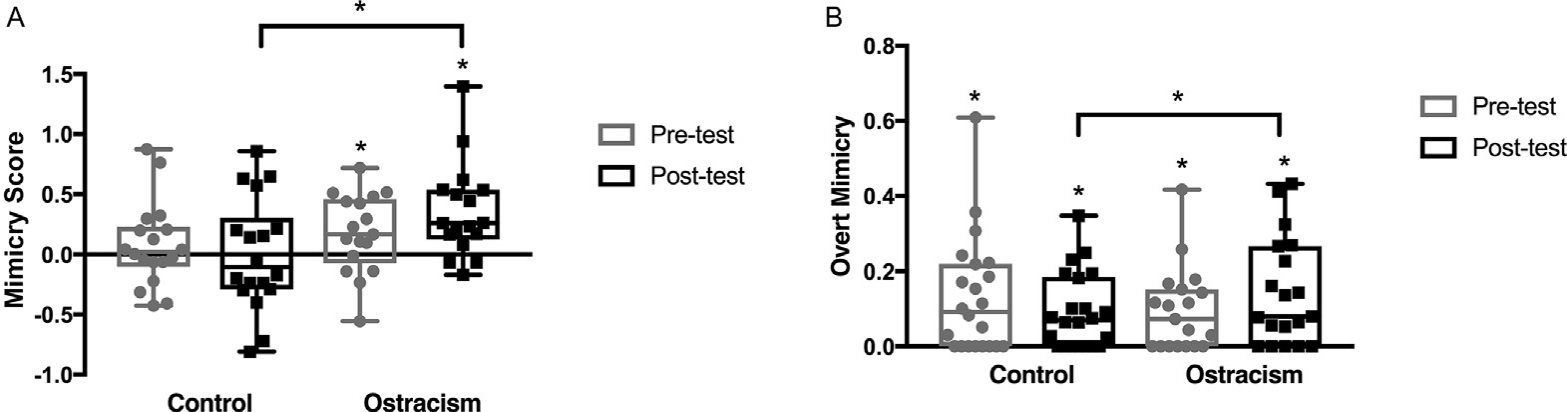
(A) Box and whisker plots of the EMG mimicry scores at pretest and posttest in the Ostracism and Control groups. (B) Box and whisker plots of the overt mimicry scores at pretest and posttest in the Ostracism and Control groups. The horizontal line within the box indicates the median, the boundaries of the box indicate the 25th and 75th percentiles, and the whiskers indicate the highest and lowest values. The circles and squares represent the individual data points. *p < .05.

To investigate the effect of condition while controlling for pretest mimicry levels, we conducted an analysis of covariance (ANCOVA) on the posttest mean mimicry scores with condition (Ostracism vs. Control) as a between-participant factor and pretest mean mimicry scores as a covariate. ANCOVA was used because it has the highest statistical power and is the recommended analysis approach in this kind of pretest–posttest design (van Breukelen, 2006, Vickers, 2001). This analysis demonstrated a significant main effect of condition, *F*(1, 32) = 4.812, *p* = .036, *η*_p_^2^ = .131. The toddlers in the Ostracism condition showed greater facial mimicry at posttest (controlling for the level of mimicry at pretest) than the toddlers in the Control condition (see Fig. 4A). The mean posttest mimicry score in the Ostracism condition was significantly different from zero, *t*(16) = 3.770, *p* = .002, but the mean mimicry score in the Control condition was not, *t*(17) = 0.006, *p* = .995. There was also a significant effect of pretest, *F*(1, 32) = 4.289, *p* = .047, *η*_p_^2^ = .118, indicating that mimicry at pretest was significantly related to mimicry at posttest. However, the pretest mimicry scores did not differ between the two conditions, *F*(1, 33) = 0.856, *p* = .362.

Because we did not exclude trials based on the toddlers’ facial expressions, the EMG measure of mimicry represents a mixture of overt and covert mimicry for a small subset of the toddlers. In the supplementary material, we report analyses performed on the EMG data from which we removed all trials in which the toddlers performed any of the facial actions with which they were presented (i.e., frowning, eyebrow raising, tongue protrusion, and mouth opening). These analyses also showed a main effect of condition, *F*(1, 32) = 4.414, *p* = .050, *η*_p_^2^ = .115, with the toddlers in the Ostracism condition showing greater covert facial mimicry at posttest than the toddlers in the Control condition. For more details, see the supplementary material.

### Overt mimicry

An ANCOVA on the mean overt mimicry scores at posttest with condition (Ostracism vs. Control) as a between-participant factor and pretest overt mimicry scores as a covariate also demonstrated a significant main effect of condition, *F*(1, 38) = 4.796, *p* = .035, *η*_p_^2^ = .112. The toddlers in the Ostracism condition showed greater overt facial mimicry at posttest (controlling for their level of overt mimicry at pretest) than the toddlers in the Control condition. The average overt mimicry scores at posttest were significantly different from zero in both conditions: Control, *t*(21) = 4.316, *p* ≤ .001; Ostracism, *t*(18) = 4.382, *p* ≤ .001. There was also a significant effect of pretest, *F*(1, 38) = 20.077, *p* < .001, *η*_p_^2^ = .346, indicating that overt mimicry at pretest was significantly related to overt mimicry at posttest. The pretest overt mimicry scores did not differ between the two conditions, *F*(1, 39) = 0.662, *p* = .421.

## Discussion

Here we show that observing third-party ostracism, which has previously been found to enhance young children’s affiliative tendencies (Over and Carpenter, 2009, Song et al., 2015, Watson-Jones et al., 2014), results in enhanced overt and covert facial mimicry in 30-month-old toddlers. These findings are consistent with previous work showing that 3- to 6-year-old children increase their imitative behaviors in response to observing third-party ostracism, but that measured the fidelity of imitation of object-directed actions instead of spontaneous mimicry behaviors (Over and Carpenter, 2009, Watson-Jones et al., 2014). These findings are also consistent with previous adult mimicry work (Lakin et al., 2008) and suggest that facial mimicry behavior may help to maintain affiliative bonds when cues indicating social exclusion are perceived from at least 30 months of age.

However, what is the exact mechanism through which observing these relatively subtle cues led to enhanced mimicry? One possibility is that observing the ostracism videos activated the toddlers’ goal to affiliate with others, and mimicking the models’ facial actions functioned as a means to communicate their similarity to the models (van Baaren et al., 2009). This goal to affiliate does not necessarily need to have been conscious (Bargh, Gollwitzer, Lee-Chai, Barndollar, & Trötschel, 2001). Previous research has shown that facial mimicry is influenced by social signals, such as eye contact and group membership, from an early age (de Klerk et al., 2018, de Klerk et al., 2018), and that infants’ copying behaviors tend to be rewarded with positive affect (Pawlby, 1977, Waxler and Yarrow, 1975). Therefore, regardless of the mechanisms that initially underlie it, the positive effect this early selective mimicry has on infants’ interaction partners is likely to result in mimicry behavior becoming positively associated with social affiliation (Heyes, 2013b, Heyes, 2017, Waxler and Yarrow, 1975). This association may in turn cause children to increase their mimicry behavior whenever they perceive that their social relationships may be under threat.

One may argue that because the facial action stimuli in the current study were presented on a screen rather than by a real-life model, it seems unlikely that the toddlers’ mimicry behavior would have been driven by attempts to affiliate with the model. Indeed, previous research has demonstrated that infants and toddlers demonstrate a “video deficit” and imitate actions less frequently and/or less accurately after observing them on a screen versus live (e.g., Nielsen et al., 2008, Barr and Hayne, 1999). However, the video deficit in imitation decreases with age (Barr & Hayne, 1999), and by 30 months of age toddlers show a significant amount of imitation even when stimuli are presented on a screen (Hayne, Herbert, & Simcock, 2003). In addition, as mentioned above, it might not be necessary for the toddlers to have a conscious goal to affiliate but rather that they have previously associated mimicry with an increase in social affiliation and that their motivation to affiliate was heightened after observing the third-party ostracism. If enhanced mimicry is an automatic consequence of having the goal to affiliate, it might not matter whether or not there is an opportunity for real-life interaction. Nevertheless, it would be interesting for future research to investigate whether the effects of observing third-party ostracism on facial mimicry are stronger when more naturalistic, real-life interactions are involved.

Another possibility is that the ostracism primes induced mild anxiety, which either directly enhanced the children’s goal to affiliate, and consequently their mimicry behaviors, or may have had a more indirect effect by altering the way in which the toddlers processed the models’ actions. It has been suggested that observing third-party ostracism may lead to an increase in arousal that in turn causes enhanced attention to any subsequent stimuli, both social and nonsocial (Heyes, 2017). The current study did not include a nonsocial mimicry condition; therefore, we cannot exclude the possibility that the toddlers in the Ostracism condition would have also mimicked a nonsocial stimulus (e.g., movements performed by a robot) to a greater extent than the toddlers in the Control condition. However, we did not find any evidence to suggest that the toddlers in the Ostracism condition paid more attention to the stimuli given that there were no differences in the number of included trials between the two groups. In addition, because only trials in which the toddlers attended to the actions were included in the analyses, it seems unlikely that attentional effects would have caused the differences in mimicry between the groups. Nevertheless, looking is not necessarily equivalent to attending (Aslin, 2012); therefore, we cannot completely rule out the possibility that the toddlers in the Ostracism condition may have processed the facial actions at posttest to a greater extent. Future studies that measure arousal (e.g., by measuring pupil dilation or heart rate) as well as neural activation during the observation of the ostracism cues and subsequent social and nonsocial stimuli would be able to investigate whether input modulation may underlie the increase in mimicry behaviors after observing third-party ostracism.

Finally, it has been shown that the suggestion of social exclusion can lead to a decrease in neural activation in areas supporting cognitive control (Campbell et al., 2006) and that being excluded during a game of Cyberball results in a decreased ability to inhibit automatic responses (Otten & Jonas, 2012). Therefore, another possibility is that the mimicry behaviors of the toddlers in the Ostracism condition were enhanced at posttest because there was less output modulation, that is, less control over the extent to which the activated motor representations were allowed to influence overt responses (Heyes, 2013a).

Note that these explanations are not mutually exclusive and that several mechanisms could have played a role in modulating toddlers’ facial mimicry responses in the current study. Although typically the goal to affiliate is thought to directly lead to the enhancement of mimicry behaviors (e.g., see Chartrand & Jefferis, 2003), studies with adult participants have shown that experiencing social exclusion also specifically enhances attention to and processing of subsequent social, but not nonsocial, stimuli (DeWall et al., 2009, Gardner et al., 2000, Gardner et al., 2005). Thus, one possibility is that the toddlers’ goal to affiliate in the Ostracism condition specifically led them to pay greater attention to the models’ facial actions, in turn leading to greater mimicry behavior. Further research will be needed to determine the exact mechanisms underlying the effect that observing third-party ostracism has on affiliative imitation.

Because one may expect that mimicry needs to be perceptible to influence one’s social partner, we hypothesized that overt mimicry may potentially be more strongly influenced by toddlers’ affiliative motivations. However, the results from the EMG measure of mimicry and the overt imitation scores were highly similar, suggesting that overt mimicry and covert mimicry are likely to be supported by similar mechanisms and motivations. This was the case even when we performed our analyses on an EMG measure of mimicry that excluded all instances of overt mimicry (see supplementary material). These findings are consistent with the idea that the subtle mimicry that can be measured by EMG is a building block for more overt and extended matching (Moody and McIntosh, 2006, Moody and McIntosh, 2011). Furthermore, these findings suggest that the transition from covert mimicry to overt mimicry constitutes a quantitative change rather than a qualitative change, where the mimicry becomes overtly visible whenever the activation of the motor representation in the mimicker reaches a certain threshold.

One thing to note about the EMG data is that the activation over the currogator and frontalis muscle region during the observation of frowning and eyebrow raising looked relatively similar (see Supplementary Fig. 1). Given that surface EMG electrodes are thought to measure broad nonselective firing of aggregates of motor units of muscle groups underlying and near the electrode sites (Lawrence & De Luca, 1983), this might not be very surprising. In addition, it is important to bear in mind that the toddlers observed dynamic stimuli in which the models moved their eyebrows from their normal position upward and back to normal in the eyebrow-raising trials and from normal to frowning and back during the frowning trials. Thus, both actions involved vertical motion of the eyebrows. Given that the frontalis provides antagonistic pull against the corrugator (Flowers & Smith, 2010) and the corrugator has been shown to be involved in elevating parts of the brow (Isse & Elahi, 2001), it is possible that both the corrugator and frontalis muscle regions were involved in the mimicry of both the eyebrow actions. Alternatively, it could be the case that some toddlers responded with a complementary fearful facial expression in response to observing the frowning stimulus. This would not be unprecedented given that a previous study by Geangu et al. (2016) found that 3-year-old children showed frontalis activation during the observation of angry faces (see Moody et al., 2007, for similar findings with adult participants). Future research will be needed to differentiate between these possibilities, for example, by measuring mimicry of both static and dynamic stimuli.

To summarize, our results demonstrate that facial mimicry is modulated by observing third-party ostracism during toddlerhood. These findings are consistent with previous studies in which 3- to 6-year-old children showed higher fidelity imitation of object-directed actions after observing third-party ostracism (Over and Carpenter, 2009, Watson-Jones et al., 2014) and suggest that both types of imitation (high-fidelity imitation of object-directed actions and mimicry) are related to social affiliation (Over & Carpenter, 2013). Furthermore, these findings show that this effect of third-party ostracism on affiliative imitation is present from at least 2.5 years of age. Although the exact mechanism underlying this increase in facial mimicry will need to be investigated in future studies, these findings are consistent with social affiliation accounts of mimicry and suggest that facial mimicry may play a key role in supporting and maintaining affiliative bonds when toddlers perceive the risk of social exclusion.

## Footnotes

1 These 6 toddlers also wore the EMG sensors but did not have a sufficient number of valid trials at pretest and/or posttest to be included in the EMG analyses. However, they did have a sufficient number of valid trials to be included in the overt mimicry analyses.

2 We previously demonstrated that infants show mimicry of these facial action stimuli from at least 4 months of age (de Klerk et al., 2018). At that visit, we also presented infants with hand actions but did not find any evidence for hand mimicry. Therefore, the hand action stimuli were not included in any subsequent visits, but this clarifies the presence of the models’ hands in the videos.

3 Note that Myon uses a floating ground. This means that it records the difference in electrical potential between two electrodes without reference to a ground electrode.

4 Our analysis approach was planned a priori and followed our previous work (de Klerk et al., 2018, de Klerk et al., 2019, 2019). We did not use baseline-corrected EMG values in these analyses. If we define mimicry as a relative pattern of muscle activation in which corresponding facial muscles are activated to a greater extent than noncorresponding facial muscles, it seems unnecessary to perform a baseline correction. Instead, by transforming the EMG activity to *z* scores and calculating mimicry scores, we can measure infants’ tendency to selectively activate the corresponding facial muscles to a greater degree than the noncorresponding facial muscles during the observation of facial actions.
