# Supplementary material for: Observing third-party ostracism enhances facial mimicry in 30-month-olds
Source: J Exp Child Psychol. 2020 Aug;196:104862. doi: 10.1016/j.jecp.2020.104862 (PMC7262587; doi:10.1016/j.jecp.2020.104862)
Supplement: Supplementary data 1 [file mmc1.docx]

**Supplementary materials**

*
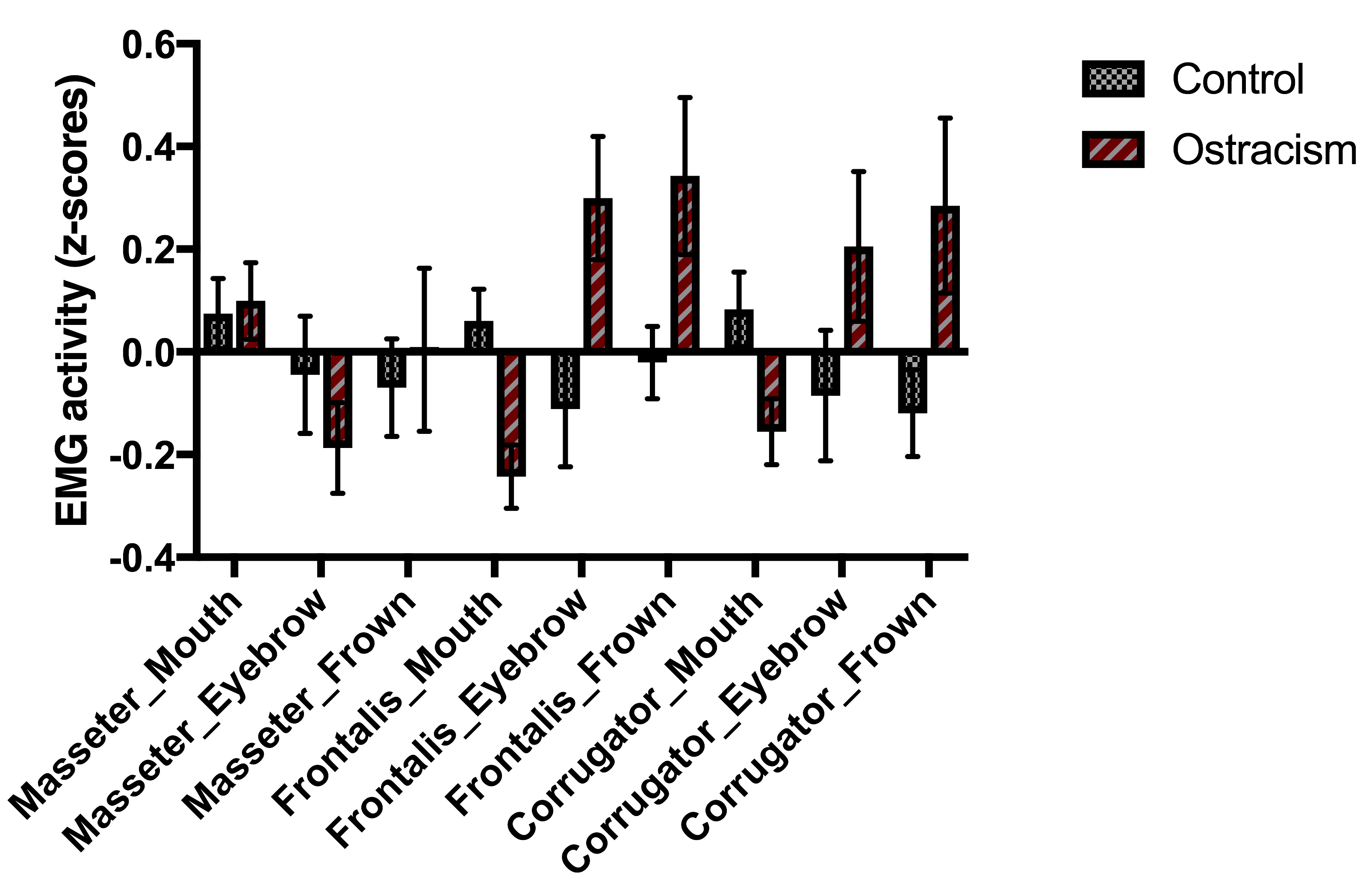
*

*Supplementary Figure 1.* Mean EMG-activity (z-scores) over the masseter, frontalis, and corrugator region during the observation of mouth actions, eyebrow raising, and frowning at post-test in the Ostracism and Control group. Error bars indicate 1 SEM.

**Covert mimicry only**

Because we did not exclude trials based on the toddlers’ facial expressions, in the main analyses reported in the paper the EMG measure of mimicry included a mixture of overt and covert mimicry for a small subset of the children. Here we report analyses on the EMG data from which we removed all trials in which the toddlers performed any of the facial actions that they were presented with (i.e. frowning, eyebrow raising, tongue protrusion, and mouth opening). An analysis of covariance (ANCOVA) on the post-test covert mimicry scores with Condition (Ostracism vs. Control) as between subject factor, and pre-test covert mimicry scores as a covariate, demonstrated a significant main effect of Condition, *F* (1, 32) = 4.141, *p* = .050, *η_p_^2^* = .115 (see Supplementary Figure 2). The effect of pre-test was not significant, *F* (1, 32) = 1.386, *p* =.248, *η_p_^2^* = .042. Only the toddlers in the Ostracism condition showed significant covert mimicry at post-test, *t*(16)= 3.219, *p*=.005, while the toddlers in the Control condition did not, *t*(17)= -.212, *p*=.834. The mimicry scores reported in the paper and these mimicry scores from which all instances of overt mimicry were removed were highly correlated, pre-test: *r*(33)=.724, *p*<.001, post-test: *r*(33)=.892, *p*<.001.

**
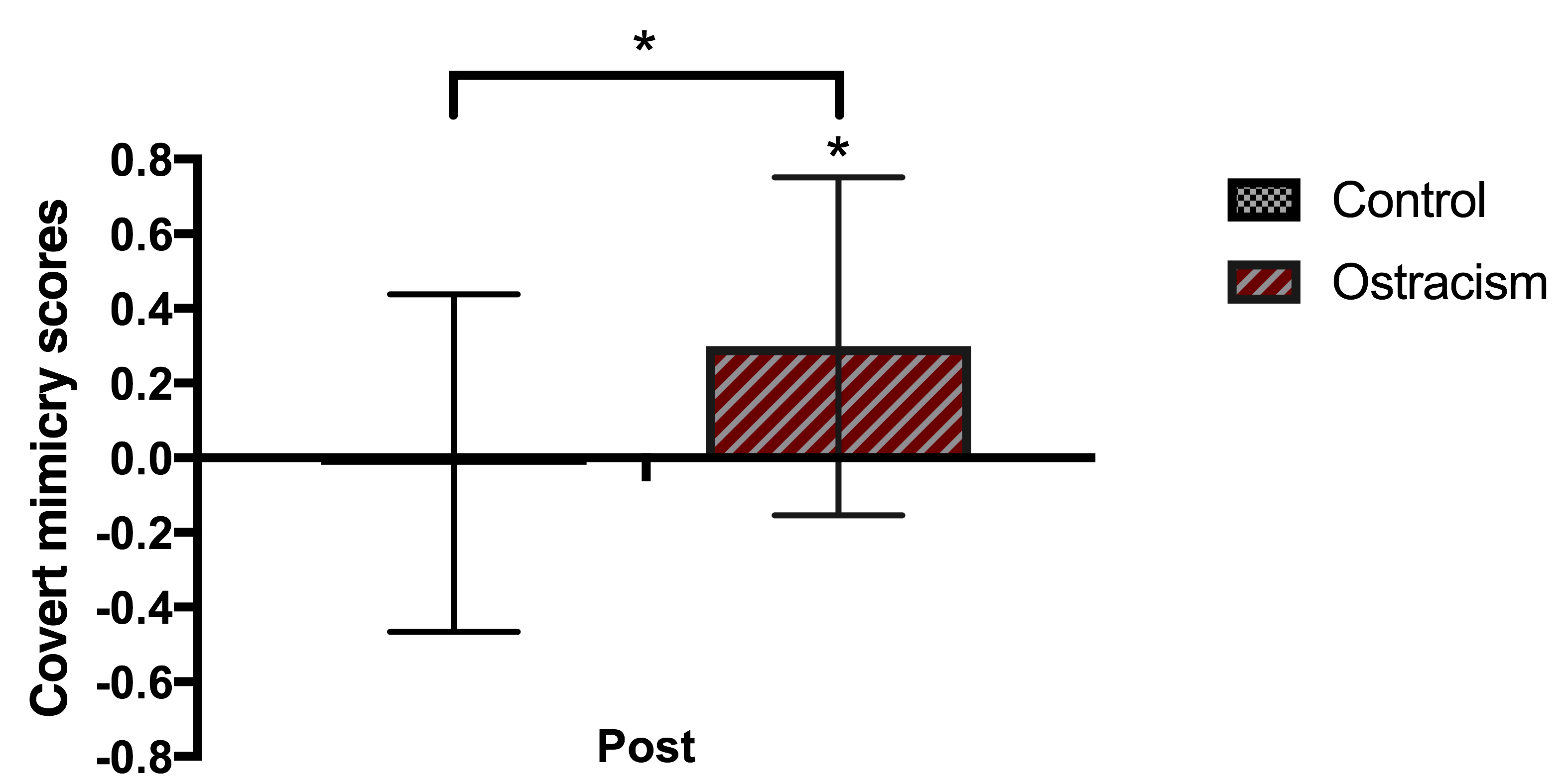
**

*Supplementary Figure 2.* Covariate-adjusted mean covert EMG mimicry scores at post-test in the Ostracism and Control group. Error bars indicate standard deviations. * *p* ≤ .05.
